# Supplementary material for: Surface Microbiome Profiling of Dental Elevators Using Third‐Generation Sequencing: Implications for Infection Control in Dental Practice
Source: Microbiologyopen. 2025 Dec 8;14(6):e70178. doi: 10.1002/mbo3.70178 (PMC12685768; doi:10.1002/mbo3.70178)
Supplement: Supplementary file 1 — customer_backup. [file MBO3-14-e70178-s001.zip › customer_backup/customer_backup/taxa_summary/krona/samples/M10.Krona.html]

Javascript must be enabled to view this page.

members
magnitude
magnitudeUnassigned

M10.krona

57454

57454

1

1

1

1

1

1

888

427

427

126

22

22

104

104

301

4

4

208

10

7

25

166

7

7

37

31

6

45

45

26

18

18

18

18

1

1

1

1

7

1

1

1

1

1

1

3

2

2

1

1

2

2

1

1

424

58

58

58

1

2

7

20

13

7

8

177

171

156

156

15

15

6

1

1

5

1

1

3

1

1

1

1

3

3

3

2

1

11

11

11

2

3

5

1

20

7

7

3

1

3

13

9

3

6

4

4

5

5

3

3

2

2

149

3

1

1

2

2

72

47

47

1

1

14

2

12

1

1

9

3

1

5

3

3

3

60

2

2

25

25

28

7

21

5

4

1

11

11

11

9

2

2

1

1

1

1

7

7

7

7

2

2

2

2

2

1

1

1

1

1

1

781

781

781

752

3

3

749

595

154

29

29

6

23

29

29

29

28

28

28

1

1

1

1

1

1

1

1

1

54

36

12

12

8

8

2

1

1

1

1

1

1

24

10

10

10

7

6

6

1

1

7

1

1

6

2

4

18

18

18

18

18

1

1

1

1

1

1

101

79

79

79

5

5

74

72

2

1

1

1

1

1

21

21

1

1

1

20

20

20

16

13

13

13

13

13

3

3

3

3

3

280

82

82

82

82

82

198

198

32

32

32

73

25

25

17

17

22

22

9

9

93

93

93

22

7

7

7

7

7

15

15

15

15

15

207

207

207

207

207

101

106

201

3

3

3

3

3

198

22

21

21

9

12

1

1

1

176

176

176

176

4348

197

4

4

2

2

1

1

1

1

3

3

3

3

131

1

1

1

7

2

2

5

5

74

39

39

1

1

1

1

2

1

1

31

7

21

3

49

1

1

15

13

2

7

4

3

26

26

2

2

1

1

1

1

46

46

6

6

12

8

1

3

1

1

2

2

25

1

3

8

7

1

1

4

1

1

1

1

10

10

1

1

2

1

1

3

3

4

4

4151

2318

1

1

1

60

3

3

52

1

51

5

5

1019

97

97

92

65

27

830

22

123

685

499

2

2

18

18

345

345

133

133

1

1

181

181

1

180

558

500

500

53

1

14

27

11

5

1

3

1

1717

1

1

1

8

3

3

5

5

28

28

28

1671

85

85

245

8

7

230

1341

1261

80

2

2

2

7

7

7

3

3

3

1

2

1

1

1

1

26

26

26

26

2

2

2

2

9

9

2

1

1

7

7

1

1

1

1

1

1

1

1

73

1

1

1

72

1

1

71

71

1

1

1

1

1

1

35498

26082

40

40

40

40

1

1

1

1

155

155

7

7

148

148

34

34

34

1

1

32

19508

19508

235

16

10

158

3

1

41

1

5

20

3

17

2

2

10

8

2

46

1

2

42

1

147

5

142

2269

29

44

1759

423

14

1830

160

1669

1

16

16

14

14

222

9

213

55

55

2

2

9

9

82

82

6

6

7

7

72

72

1

1

52

1

11

1

32

1

6

14411

14264

143

4

1739

919

81

81

210

17

193

180

180

13

13

312

312

95

13

81

1

28

28

554

12

12

192

192

301

301

49

49

266

266

266

73

73

50

45

5

23

23

507

28

28

28

76

76

76

403

403

403

437

145

145

145

9

9

9

252

252

252

31

31

31

3588

2471

62

62

5

5

18

18

67

63

4

1

1

31

31

1120

24

22

448

382

85

159

528

528

9

9

8

8

97

27

23

47

525

135

390

32

32

32

64

64

64

1006

6

6

130

71

59

12

12

320

196

89

35

50

7

43

454

370

84

1

1

33

33

7

2

2

5

1

4

8

8

8

8258

62

36

1

1

33

33

2

2

26

26

1

1

6

1

13

1

1

1

1

1337

397

33

33

1

1

362

1

9

350

2

1

1

940

671

671

140

140

9

9

65

65

40

40

15

15

1017

159

159

45

114

858

858

858

1

1

1

1

13

13

13

1

1

9

1

1

600

114

114

114

6

6

6

243

243

243

237

237

237

6

6

6

5

1

5150

2

2

2

321

319

319

2

2

4406

4404

238

25

3496

2

539

101

1

2

2

2

3

3

3

418

84

32

1

51

35

35

279

279

8

8

1

1

1

1

9

4

3

2

1

1

72

72

72

72

1158

1

1

1

1

1157

206

206

117

10

79

951

711

702

9

69

69

171

139

32

2

2

2

2

2

2

661

661

661

661

661

661

14360

14360

19

19

1

1

7

4

3

11

11

31

25

24

7

6

11

1

1

6

6

6

14284

10346

20

20

30

30

515

515

9781

9781

808

1

1

807

1

15

62

729

1

1

1

71

71

11

25

35

2368

2368

1

2

2097

1

147

96

23

1

20

19

19

1

1

640

121

121

15

15

315

315

1

1

108

52

7

49

80

10

6

64

30

30

30

1

1

1

1

25

3

3

1

2

10

10

10

12

12

5

1

1

2

3

1

1

1

1

1

1
